# Assaying Carcinoembryonic Antigens by Normalized Saturation Magnetization

**DOI:** 10.1186/s11671-015-0964-6

**Published:** 2015-07-03

**Authors:** Kai-Wen Huang, Jen-Jie Chieh, Jin-Cheng Shi, Ming-Hsien Chiang

**Affiliations:** Institute of Electro-Optical Science and Technology, National Taiwan Normal University, 116 Taipei, Taiwan; Department of Surgery and Hepatitis Research Center, National Taiwan University Hospital, 100 Taipei, Taiwan; Graduate Institute of Clinical Medicine, National Taiwan University, 100 Taipei, Taiwan; Department of Anatomy and Cell Biology, National Taiwan University, 100 Taipei, Taiwan

**Keywords:** Magnetic immunoassays, Saturation magnetization, Magnetic clusters, Carcinoembryonic antigen, Biofunctionalized magnetic nanoparticles

## Abstract

Biofunctionalized magnetic nanoparticles (BMNs) that provide unique advantages have been extensively used to develop immunoassay methods. However, these developed magnetic methods have been used only for specific immunoassays and not in studies of magnetic characteristics of materials. In this study, a common vibration sample magnetometer (VSM) was used for the measurement of the hysteresis loop for different carcinoembryonic antigens (CEA) concentrations (*Φ*_CEA_) based on the synthesized BMNs with anti-CEA coating. Additionally, magnetic parameters such as magnetization (*M*), remanent magnetization (*M*_R_), saturation magnetization (*M*_S_), and normalized parameters (Δ*M*_R_/*M*_R_ and Δ*M*_S_/*M*_S_) were studied. Here, Δ*M*_R_ and Δ*M*_s_ were defined as the difference between any Φ_CEA_ and zero *Φ*_CEA_. The parameters *M*, Δ*M*_R_, and Δ*M*_S_ increased with *Φ*_CEA_, and Δ*M*_S_ showed the largest increase. Magnetic clusters produced by the conjugation of the BMNs to CEAs showed a Δ*M*_S_ greater than that of BMNs. Furthermore, the relationship between Δ*M*_S_/*M*_S_ and *Φ*_CEA_ could be described by a characteristic logistic function, which was appropriate for assaying the amount of CEAs. This analytic Δ*M*_S_/*M*_S_ and the BMNs used in general magnetic immunoassays can be used for upgrading the functions of the VSM and for studying the magnetic characteristics of materials.

## Background

**Fig. 1:**
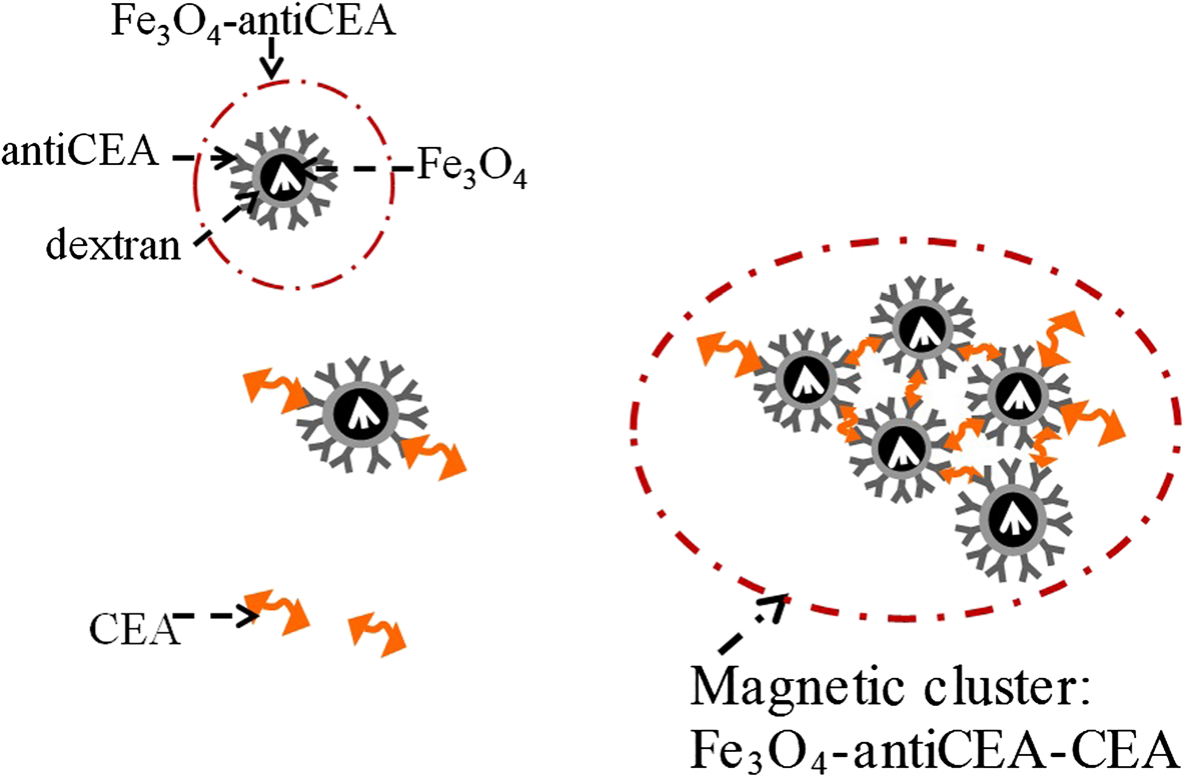
A scheme of CEAs, Fe_3_O_4_-anti-CEA, and Fe_3_O_4_-anti-CEA-CEA. Some Fe3O4-anti-CEAs become as magnetic cluster, Fe3O4-anti-CEA-CEA, after binding to CEA antigen

Magnetic nanoparticles interest researchers because of their potential applications in biomedicine, such as protein purification [1], magnetofection [2], tomographic imaging [3], magnetic resonance imaging [4–6], magnetic immunoassays [7, 8], tumor diagnosis [9], and hyperthermia therapy [10]. In magnetic immunoassays, magnetic nanoparticles are first biofunctionalized with antibodies to obtain biofunctionalized magnetic nanoparticles (BMNs), which are then dissolved in solutions to form magnetic reagents. To assay a biotarget, a magnetic reagent is mixed with a sample solution containing the biotarget. The conjugation of BMNs with the biotarget produces magnetic clusters because of molecular interaction (Fig. 1), and the magnetic properties of the reagent changes. Biological samples, unconjugated BMNs, and magnetic clusters of conjugated biotargets show a negligible magnetic background individually and differ in their magnetic characteristics. Hence, it is possible to develop magnetic immunoassays on the basis of several parameters and phenomena such as magnetic relaxation [11, 12], remanent magnetization (*M*_R_) [13, 14], saturation magnetization (*M*_S_) [15], magnetic resonance [16, 17], and alternating current (ac) susceptibility (*χ*_ac_) [8, 18–21].

In addition, because signal changes associated with the magnetic characteristics of BMNs are always small, a high-sensitivity high-critical-temperature superconducting quantum interference device (SQUID) sensor is usually used to enhance the signal-to-noise ratio and mu-metal shielding is provided to reduce environmental noise. A cryogenic biodetection system involving SQUIDs is difficult to construct.

Washing processes are sometimes required to separate magnetic clusters from reagents for measuring magnetic characteristics; however, they are time-consuming. Therefore, developing a biodetection system featuring an alternative detection mechanism and high detection sensitivity is crucial. A wash-free immunomagnetic reduction (IMR) method based on ac magnetic susceptibility reduction has been proposed [19], and various studies have demonstrated the sensitive detection of biomolecules, such as nucleic acids [20], biomarkers (for diagnosing Alzheimer’s disease) [6], alpha-fetoprotein (for detecting liver tumors) [7], and human C-reactive protein (for diagnosing inflammation) [15].

In this study, we proposed a magnetic immunoassay method based on the BMNs used in magnetic immunoassay methods, like IMR; the proposed method does not require a SQUID sensor or washing process. The method involves the use of a vibration sample magnetometer (VSM) for measuring the hysteresis loop, from which the major magnetic characteristics can be inferred, and does not require a specific magnetic instrument for magnetic immunoassays. The magnetic parameters of the hysteresis loop were studied to determine the analytic method of magnetic immunoassay. When the method is applied to magnetic immunoassays, the magnetic parameters of the analytics are determined from the hysteresis loop.

## Methods

Figure 1 shows a schematic of the clustering process involving BMNs and dextran-coated Fe_3_O_4_ nanoparticles. The procedures used for synthesizing BMNs consisting of anticarcinoembryonic antigens (anti-CEAs) coated on dextran-coated Fe_3_O_4_ nanoparticles (MF-DEX-0060, MagQu Corp., Taiwan) were similar to those used in a previous study for synthesizing dextran-coated Fe_3_O_4_ nanoparticles coated with anti-goat C-reactive protein [22]. Dextran-coated Fe_3_O_4_ nanoparticles was oxidized using NaIO_4_ to create aldehyde groups (−CHO), and dextran reacted with the antibodies of anti-CEAs (10C-CR2014M5, Fitzgerald, MA, USA) through −CH = N- to covalently conjugate the antibodies of anti-CEAs. After magnetic separation, the unbound antibodies were separated from conjugated BMNs consisting of dextran-coated Fe_3_O_4_ nanoparticles coated with anticarcinoembryonic antigens (Fe_3_O_4_-anti-CEAs). Subsequently, a reagent was synthesized by dissolving the BMNs in phosphate-buffered saline. The biotargets were carcinoembryonic antigens (CEAs; 30-AC30, Fitzgerald, MA, USA). These antigens are typically used as a tumor marker for colorectal cancers, which are caused by uncontrolled cell growth in the colon or rectum [23] and are the second leading cause of cancer death in adults worldwide [24].

**Fig. 2:**
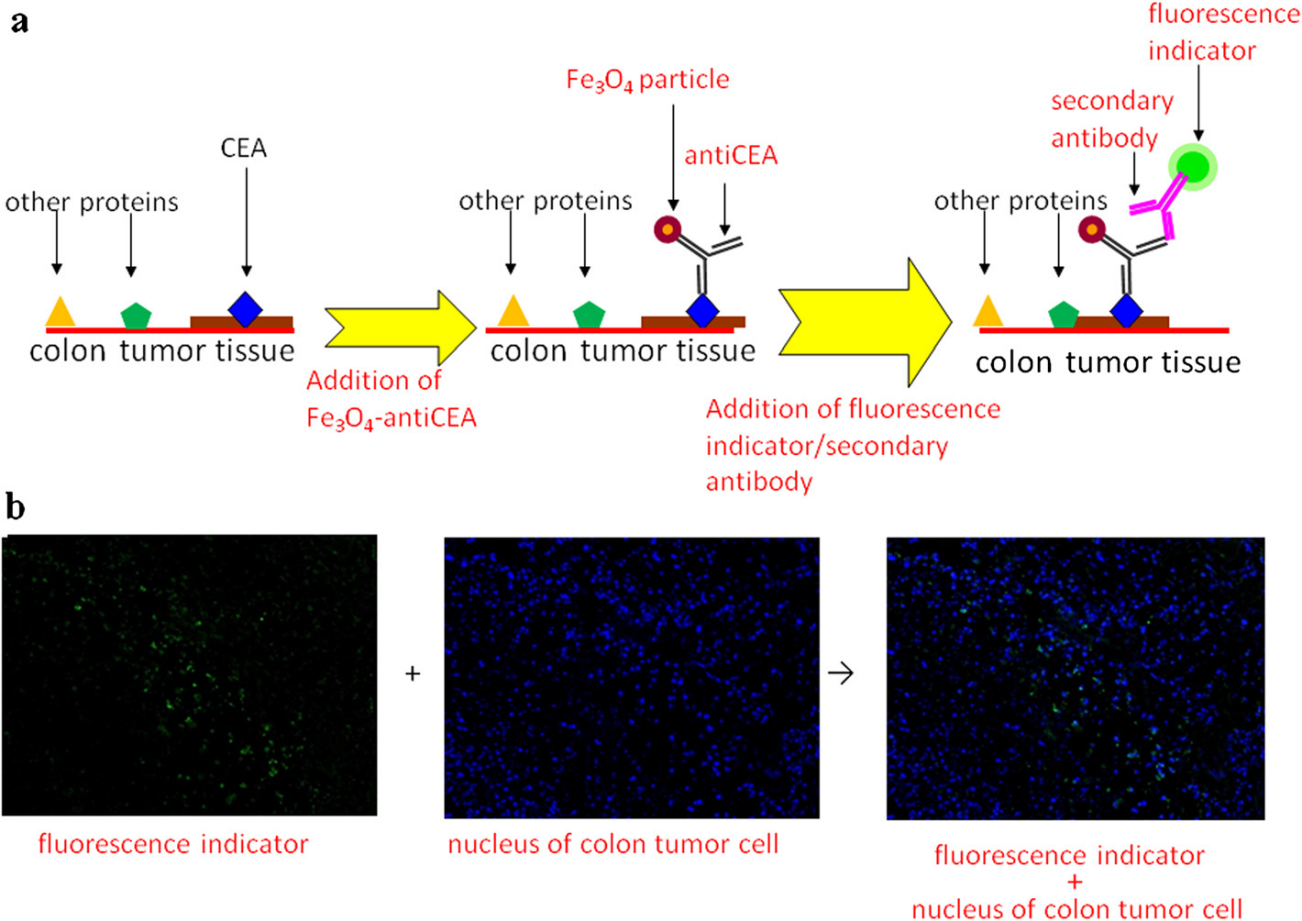
The stain of colon tumor tissue using the Fe_3_O_4_-anti-CEA reagent. **a** The stain process. **b** The fluorescence images with *blue* and *green colors*, representative of the nucleus of a colon tumor cell and the fluorescence indicator on BMNs

The mean value of the hydrodynamic diameter of the BMNs was 40.8 nm, as detected through dynamic laser scattering (Nanotrac 150, Microtrac, PA, USA). The conjugation capability of BMNs was verified by tissue staining. The colon tumors induced on the backs of mice were sampled to form paraffin-embedded sections. Figure 2a shows the process of staining the colon tumor tissue with BMNs. First, the sections of the colon tumors were immersed in the Fe_3_O_4_-anti-CEA reagent. Consequently, a secondary antibody conjugated to a fluorescent indicator (goat anti-rabbit IgG antibody, Millipore, USA) was added. Here, the binding occurred because the fluorescent indicator with an isothiocyanate reactive group was reactive toward nucleophiles containing amine and sulfhydryl groups on the protein [25]. Because of conjugation between the secondary antibodies and anti-CEA antibodies, the fluorescent indicators were bound to the BMNs on the tissue. Both the tissue and fluorescent indicators of the BMNs were obtained through fluorescence microscopy (IX70, Olympus, Japan).

In assaying the CEAs, 40 μL of the Fe_3_O_4_-anti-CEA reagent with a saturation magnetization of 0.07 emu/g was mixed with 60 μL of a CEA solution with a CEA concentration (*Φ*_CEA_) in the range from 0 to 10 ppm. To verify the formation of magnetic clusters during the assay, the effective relaxation time *τ*_eff_(*t*) was monitored. This was because the presence of magnetic clusters would increase *τ*_eff_. Furthermore, *χ*_ac_(*t*) can be expressed as follows [26, 27]:

1 $$ {\chi}_{\mathrm{ac}}(t) = {\chi}_{\mathrm{ac},0}\left\{1/{\left[1 + {\left(w{\tau}_{\mathrm{eff}}(t)\right)}^2\right]}^{1/2}\right\} $$

Here, *χ*_ac,0_ is *χ*_ac_ of the Fe_3_O_4_-anti-CEA reagent initially mixing with the CEA solution, and *ω* is the angular frequency. Therefore, *τ*_eff_ can be obtained by substituting Δ*χ*_ac_, defined as *χ*_ac,0_ − *χ*_ac_, in Eq. (1). The test materials were the Fe_3_O_4_-anti-CEA reagent and a CEA solution with a *Φ*_CEA_ of 10 ppm. The complete experiment process first involved the measurement of the hysteresis loop for only the Fe_3_O_4_-anti-CEA reagent by using the VSM (Model Hystermag, MagQu Corp., Taiwan). Subsequently, *χ*_ac_ for the mixture of the reagent and the CEA solution was measured continuously during the entire assay period by using an analyzer (χ_ac_Pro-E101, MagQu Corp., Taiwan). After the assay, the mixture was again measured using the VSM.

**Fig. 3:**
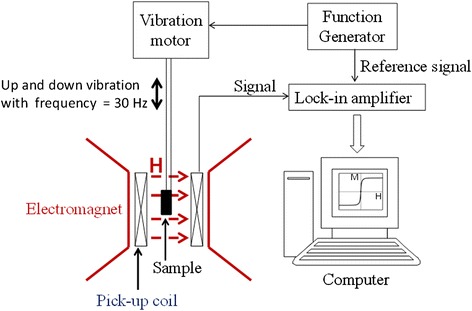
The measurement scheme of the hysteresis loop using a VSM

For a *Φ*_CEA_ of 10 ppm, the formation of magnetic clusters in the assay of the CEAs was verified by measuring *χ*_ac_ along with the hysteresis loop during the assay period. For all the other CEA concentrations (0, 0.01, 0.5, 1, 2.5, and 5 ppm), only the hysteresis loop was measured. Figure 3 shows a schematic of the measurement of the hysteresis loop, which expresses the magnetization *M* as a function of the applied field *H*. An electromagnet that provided a maximum *H* of 1.0 T was used to determine *M*, *M*_R_, and *M*_S_. The sample was vibrated with a frequency of approximately 30 Hz by using an oscillating device. The magnetic signal was then detected using a second-order gradient pickup coil. In addition to characterizing the variation of Δ*M*_R_ or Δ*M*_S_ with *Φ*_CEA_, the relationship between Δ*M*_R_/*M*_R_ or Δ*M*_S_/*M*_S_ and *Φ*_CEA_, which represented the merit function of the CEA amount, was determined.

## Results and Discussion

Figure 2b shows BMPs conjugated to the CEAs on the tumor tissue. The blue and green colors represent the nucleus of a colon tumor cell and the fluorescent indicator, respectively. Here, the excitation/emission wavelengths of the observed green and blue colors were 495 nm/519 nm and 358 nm/461 nm, respectively. Superposing these two images shows that the blue and green spots are located in close proximity, indicating that the BMPs were bound to colon tumor cells. The proximity of the blue and green spots also confirms the bioconjugation capability of the BMNs.

**Fig. 4:**
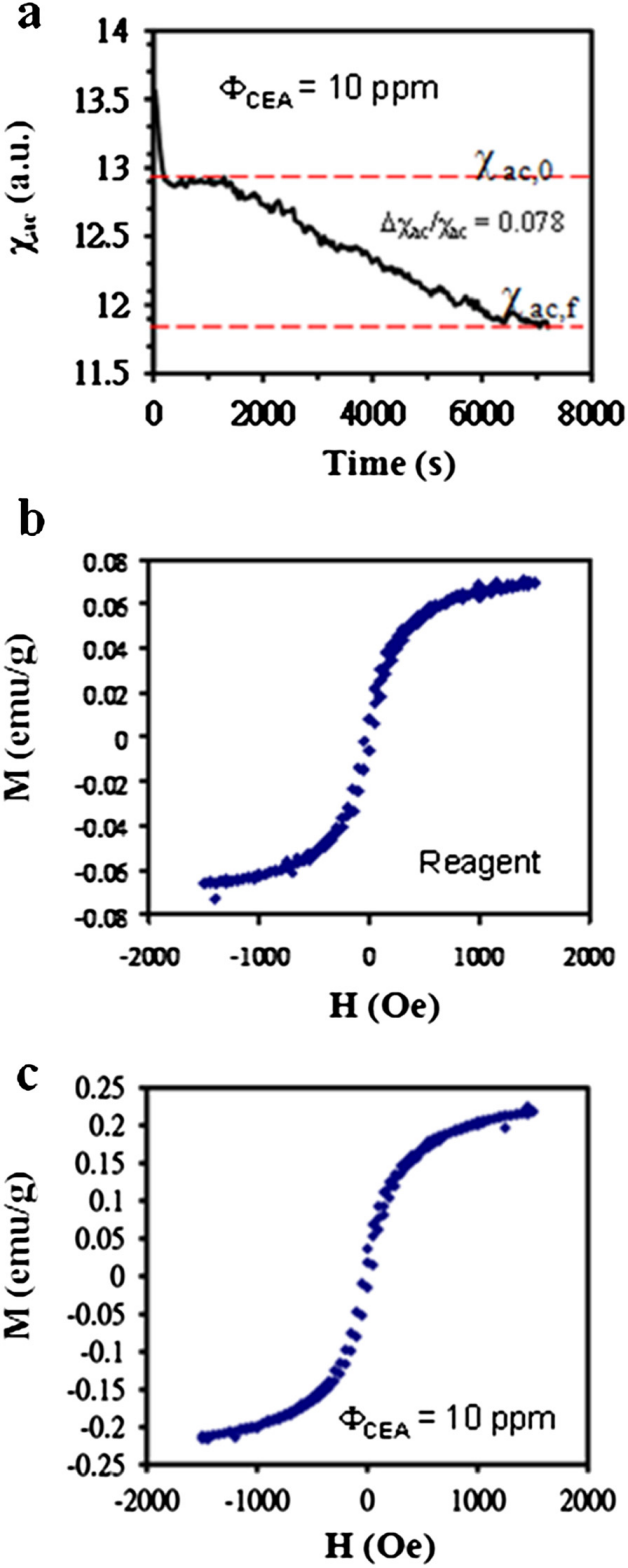
The magnetic measurements of χ_ac_ and the hysteresis loop for mixing 40 μL of the Fe_3_O_4_-anti-CEA reagent with 60 μL and 10 ppm of a CEA solution. **a** The dynamic measurement of χ_ac_ with time. **b** Before and **c** after the measurement of χ_ac_, the measurement of the hysteresis loop for only the Fe_3_O_4_-anti-CEA reagent as well as the mixture of the same reagent and the CEAs

Figure 4a shows that *χ*_ac_ was initially constant and that it subsequently decreased with time and reached a steady value. These stages corresponded to the preconjugation, conjugation, and postconjugation period, in which the reference is to the conjugation between BMNs and CEAs. In the immunomagnetic reduction (IMR) assay [8, 18–21], the normalized parameter Δ*χ*_ac_/*χ*_ac_ (the IMR parameter) depends on *Φ*_CEA_. Here, Δ*χ*_ac_ is the difference in *χ*_ac_ between preconjugation *χ*_ac,0_ and postconjugation *χ*_ac,f_.

In addition to the *χ*_ac_ measurement, typical hysteresis loops of the Fe_3_O_4_-anti-CEA reagent before the assays and the mixture of the same reagent and the CEAs after assaying 10 ppm of CEAs are separately shown in Fig. 4b. The parameter *M*s for the reagent was equal to 0.07 emu/g at 0.15 T and near the saturation field, and *M*s was enhanced to 0.23 emu/g after the conjugation.

**Fig. 5:**
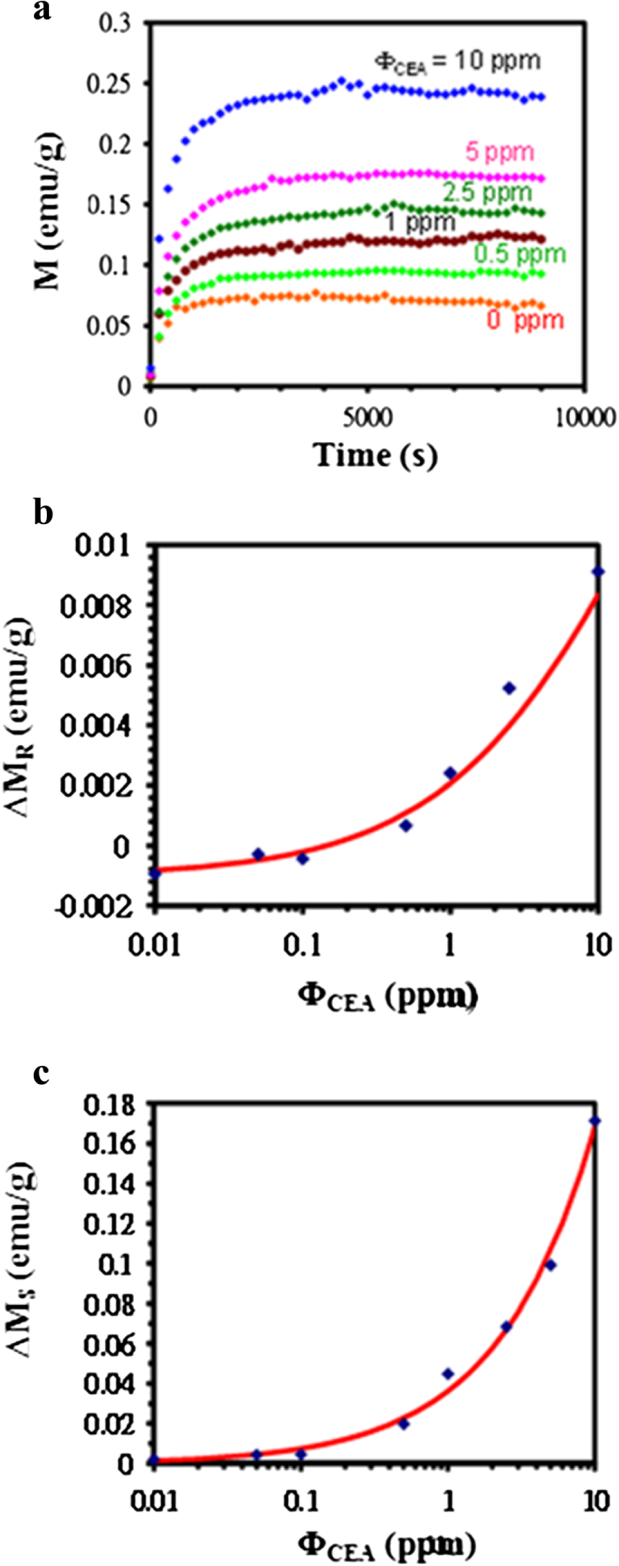
The dependence of magnetic characteristics of on Φ_CEA_ from 0.01 to 10 ppm. **a** One part of the hysteresis loop, the M variation with H, under different Φ_CEA_. **b** ΔM_S_ and **c** ΔM_R_ as a function of Φ_CEA_

One part of the hysteresis loops for various *Φ*_CEA_ values is shown in Fig. 5a. For all *Φ*_CEA_ values, *M* rapidly increased with an increase in *H* from 0 to 1000 Oe, and then gradually reached *M*_S_. Furthermore, for each *H*, *M* (including *M*_S_) increased with *Φ*_CEA_. From the hysteresis loops, both Δ*M*_R_ at zero *H* and Δ*M*_S_ at the maximum *H*, defined as the difference between Δ*M*_R_ and Δ*M*_S_ between any *Φ*_CEA_ and zero *Φ*_CEA_, also increased with *Φ*_CEA_, as depicted in Fig. 5b, c. Each of the parameters Δ*M*_R_ and Δ*M*_S_ increased to 0.009 and 0.17 emu/g for a *Φ*_CEA_ of 10 ppm.

**Fig. 6:**
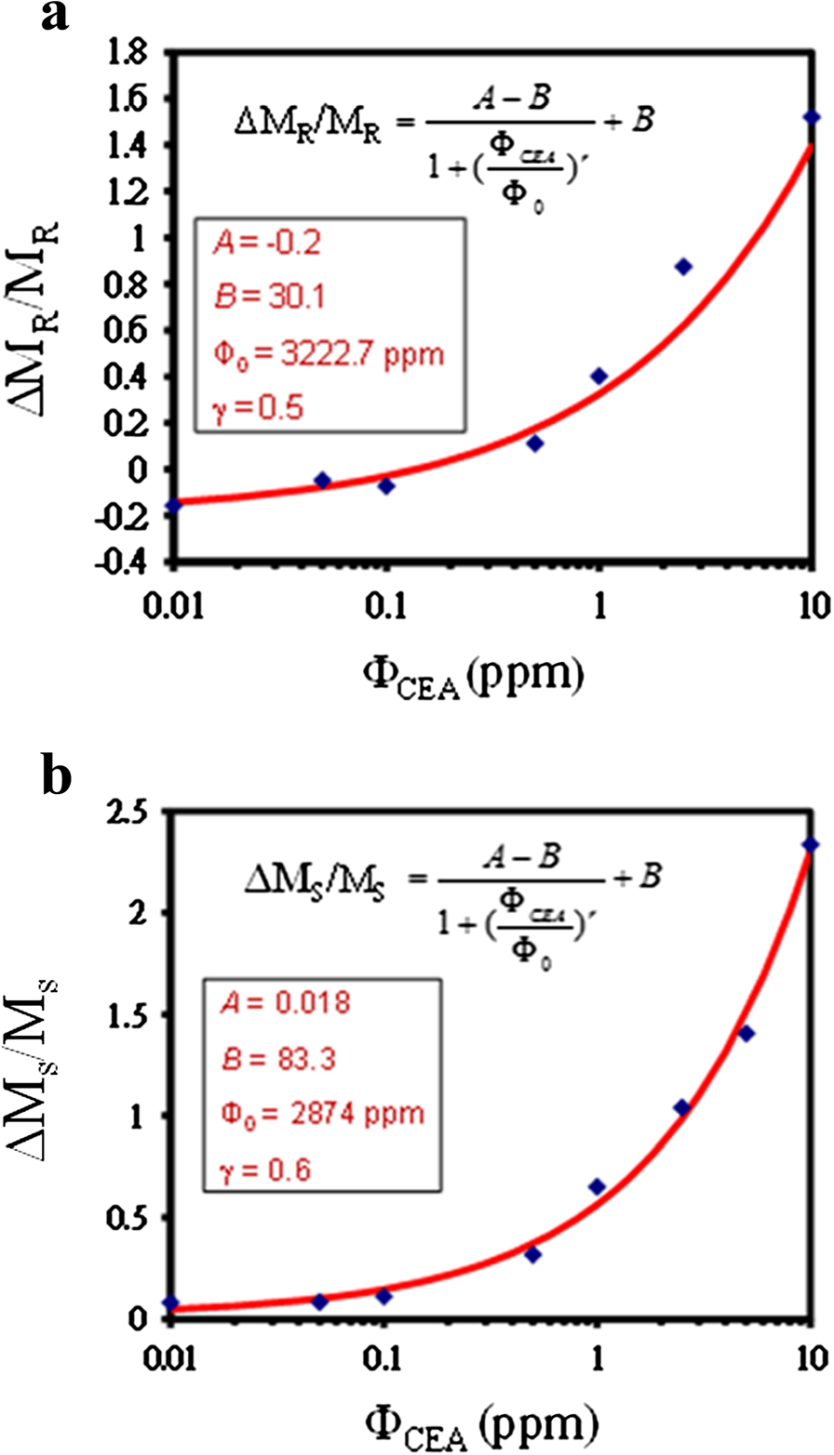
The dependence of normalized ΔM_x_/M_x_ on Φ_CEA_ from 0.01 to 10 ppm. **a** ΔM_R_/M_R_ and **b** ΔM_S_/M_S_ as a function Φ_CEA_

To quantify the detected *Φ*_CEA_ amount and to improve the capability of distinguishing the small measured values of *M*, the parameters Δ*M*_R_/*M*_R_ and Δ*M*_S_/*M*_S_ were used. In addition to the increase in the variation of Δ*M*_R_ or Δ*M*_S_ with *Φ*_CEA_, both Δ*M*_R_/*M*_R_ and Δ*M*_S_/*M*_S_, represented as Δ*M*_x_/*M*_x_, can be expressed by a characteristic logistic function *Φ*_CEA,_ as shown in Fig. 6a, b [28, 29, 19]:

2 $$ \Delta {M}_{\mathrm{x}}/{M}_{\mathrm{x}} = \left(A-B\right)/\left\{1 + {\left[\left({\varPhi}_{\mathrm{CEA}}\right)/\left({\varPhi}_0\right)\right]}^{\gamma}\right\} + B $$

where *A*, *B*, and *γ* are dimensionless quantities, and *Φ*_0_ is the dimensional concentration. The parameters *A*, *B*, *γ*, and *Φ*_0_ for the fitting curve were −0.2, 30.1, 0.5, and 3222.7 ppm for *x* = R and 0.018, 83.3, 0.63, and 2874 ppm, respectively, for *x* = S.

A comparison of Fig. 4a, b, and c shows that *χ*_ac_ decreased, and *M*, which was related to the dc magnetic susceptibility, increased after the assaying of the CEAs. The opposite variations of the ac and dc magnetic susceptibilities are attributed to the presence of magnetic clusters. The verification performed in this study was for the increase in *τ*_eff_ during conjugation, consistent with similar assays of C-reactive proteins [30]. Yang et al. [31] conducted a study on temperature-dependent immunoreaction kinetics of the BMN assay for biomarkers of colorectal cancer. They observed a gradual increase in the mean diameter of the magnetic nanoparticles from 41.53 to 45.13 nm after the reagent and CEA solution were mixed. Their results suggested the presence of magnetic clusters in the reagents. Here, the diameter of the magnetic cluster might be considerably greater than 45.13 nm, as indicated in Fig. 1. However, the magnetic clusters were confined to a limited part of the entire Fe_3_O_4_-anti-CEA reagent. Therefore, the observed increase in the mean diameter of the mixture, consisting of the Fe_3_O_4_-anti-CEA reagent and CEA solution, was small, even though individual magnetic clusters showed a considerably larger increase.

Consequently, in Fig. 5, the higher the *Φ*_CEA_ value, the larger the Δ*M*_R_ and Δ*M*_S_ values_._ However, for small values of Δ*M*_R_ or Δ*M*_S_, it is difficult to determine the *Φ*_CEA_ amount because of the small difference between Δ*M*_R_ and Δ*M*_S_. The parameter Δ*M*_R_ was scattered and negative when *Φ*_CEA_ was smaller than 0.1 ppm. The reason is that the system noise intensity was greater than the intensity of the signal for the low *Φ*_CEA_. Consequently, Δ*M*_R_/*M*_R_ or Δ*M*_S_/*M*_S_ with larger values than Δ*M*_R_ or Δ*M*_S_ was used to obtain a characteristic logistic function of *Φ*_CEA_. These relationships were identified for assaying the amount of CEAs. In particular, because of having higher values than Δ*M*_R_/*M*_R_, it is suggested that Δ*M*_S_/*M*_S_ can be used to enhance the discrimination capability of *Φ*_CEA_ in magnetic immunoassays. In Fig. 5b, c, the detection limits of Δ*M*_R_/*M*_R_ and Δ*M*_S_/*M*_S_ are 0.1 and 0.01 ppm, respectively. For the mixture of the Fe_3_O_4_-anti-CEA reagent and CEAs, if the mixing conditions such as the concentration or volume of each material can be optimized instead of the IMR condition, the detection limit can be improved for a Φ value of 0.005 ppm. This study performed a more detailed investigation compared with a previous study [32]; the investigation included validating and comparing the analysis of Δ*M*_R_/*M*_R_ and Δ*M*_S_/*M*_S_, determining the immunoassay capability of the Fe_3_O_4_-anti-CEA reagent by tissue staining, and verifying the presence of magnetic clusters through an analysis of the effective relaxation time. Moreover, the biomarker studied here was also different from that studied previously [32].

The major clinical objectives of assaying CEAs are to screen a colorectal cancer, evaluate the effect of colorectal carcinoma treatment, identify recurrences after surgical resection, and control the spread of cancer. Although a variety of developed immunoassay methodologies exist, such as enzyme-linked immunoassays [33, 34], Western blot immunoassay [35, 36], fluorescence in situ hybridization [37], and polymerase chain reactions [38], washing processes are always required to avoid inaccuracies in the optical examination of sample interference colors. This results in the immunoassays being time-consuming and requiring large manpower. In this study, the magnetic detection platform using BMNs neither depends on the color of biological samples nor requires washing. The established relationship between Δ*M*_S_/*M*_S_ and *Φ*_CEA_ followed a characteristic logistic function and was used for the determination of the CEA amount. The proposed method can be applied to the analysis of other biotargets once the relationship between Δ*M*_S_/*M*_S_ and *Φ*_biotargets_ is established.

## Conclusions

A detection mechanism was proposed to show that *M*_S_ for BMNs consisting of Fe_3_O_4_-anti-CEAs increased after conjugation with CEAs. Hysteresis loops were measured and analyzed to determine Δ*M*_R_/*M*_R_ and Δ*M*_S_/*M*_S_. Δ*M*_S_/*M*_S_ showed higher sensitivity and greater discrimination capability than Δ*M*_R_/*M*_R_ for assaying CEAs_._ Consequently, the CEA amount could be determined using the relationship between Δ*M*_S_/*M*_S_ and *Φ*_CEA_, expressed by a universal characteristic logistic function. This methodology has the potential to be used for other targets; for this purpose, magnetic reagents used in other magnetic immunoassays can be used with the VSM, and no specific instrument is required for applying the methodology to magnetic immunoassays.
